# The Role of Cardiovascular Function in Endogenous Pain Modulation

**DOI:** 10.1155/prm/8018460

**Published:** 2026-11-05

**Authors:** Joëlle Katharina Metzger, Shane James Timothy Balthazaar, Sophie Sturzenegger, Tom Edward Nightingale, Michèle Hubli

**Affiliations:** ^1^ Spinal Cord Injury Center Balgrist University Hospital University of Zurich Zurich Switzerland balgrist.ch; ^2^ Neuroscience Center Zurich (ZNZ) University of Zurich and ETH Zurich Zurich Switzerland ethz.ch; ^3^ School of Sport, Exercise and Rehabilitation Sciences University of Birmingham Birmingham UK birmingham.ac.uk; ^4^ International Collaboration on Repair Discoveries University of British Columbia Vancouver British Columbia Canada ubc.ca

**Keywords:** baroreflex sensitivity, blood pressure, conditioned pain modulation, endogenous pain modulation, heart rate variability, offset analgesia, pain-autonomic interaction

## Abstract

Structural and functional overlap between the nociceptive and cardiovascular systems establish a bidirectional coupling that supports dynamic regulation across both systems. Disruption of this coupling could contribute to impaired endogenous pain modulation in chronic pain conditions. Thus, this study aimed to investigate the relationship between endogenous pain modulation and cardiovascular function in healthy participants. Thirty participants underwent assessment of pain modulatory capacity using offset analgesia (OA; 47–48°C‐47°C) and conditioned pain modulation (CPM), with pressure pain thresholds as test stimuli and a cold pressor test (CPT) as conditioning stimulus. Resting heart rate variability (HRV) and baroreflex sensitivity (BRS) were derived from 5‐min baseline recordings of continuous electrocardiography and beat‐to‐beat blood pressure. Blood pressure reactivity during the CPT was calculated as the relative difference between peak blood pressure during the CPT and baseline. Associations between pain modulation (OA, CPM) and cardiovascular function (HRV, BRS, blood pressure reactivity) were examined. In this healthy cohort (*N* = 30), participants exhibited pronounced pain inhibitory capacity, with OA at −85.7% ± 21.8% and CPM at −19.0% ± 14.7%. Higher resting BRS was related to higher OA (rho = −0.48, *p* = 0.030) and CPM capacity (rho = −0.52, *p* = 0.021). Moreover, higher CPM capacity was associated with larger blood pressure responses in both parallel (rho = −0.48, *p* = 0.021) and sequential designs (rho = −0.49, *p* = 0.033). Increased OA capacity was linked to higher HRV (rho = −0.50, *p* = 0.030) at rest. Using a multimodal approach, these findings support a functional coupling between endogenous pain modulation and cardiovascular regulation in healthy individuals, emphasizing the role of cardiovascular adaptability in shaping pain inhibitory processes. Understanding this pain‐cardiovascular coupling may help explain interindividual variability in endogenous pain modulation in healthy individuals and might offer insight into its disruption in chronic pain conditions.

**Trial Registration:** ClinicalTrials.gov identifier: NCT06443281

## 1. Introduction

Endogenous pain modulation refers to a range of central nervous system mechanisms that modulate pain perception through descending modulatory pathways originating in the brainstem and projecting to the dorsal horn of the spinal cord. These pathways exert either inhibitory or facilitatory influences on the transmission of nociceptive input to higher centers [1]. When the balance of descending modulation shifts toward facilitation, nociceptive transmission is enhanced rather than suppressed [2, 3]. Such a facilitatory predominance and/or inhibitory deficiency of endogenous pain modulation has been linked to various chronic pain conditions (for review, see [4]) and is commonly measured as reduced conditioned pain modulation (CPM) [5, 6] or offset analgesia (OA) [7, 8]. These two measures provide complementary [9], rather than redundant, information on endogenous pain modulation since they reflect partially distinct mechanisms [10, 11]. CPM reflects endogenous pain modulation in which the perception of a noxious test stimulus is modulated by a concurrent or preceding heterotopically applied noxious conditioning stimulus. Commonly described as “pain inhibits pain,” this phenomenon is thought to reflect spatial filtering of nociceptive information [11, 12]. In contrast, OA refers to a disproportionately large reduction in perceived pain intensity evoked by a slight decrease in noxious stimulus intensity, representing temporal filtering of nociceptive information [13, 14].

Previous studies have demonstrated the clinical relevance of endogenous pain modulation by showing that deficient pain inhibition has been associated with an increased risk of postoperative pain [15–18], sleep deprivation [19], and reduced responsiveness to analgesics [20, 21]. Furthermore, diminished CPM efficacy has been reported in patients with osteoarthritis and fibromyalgia [22], with reduced CPM efficacy linked to increased fibromyalgia symptom severity, anxiety, and fatigue levels [23]. Individual differences in endogenous pain modulation, reflecting inherent variability in central nervous system processing, may also indicate susceptibility to the development of chronic pain [22, 24]. Identifying factors that determine endogenous pain modulatory capacity could thus enhance our understanding of its underlying mechanisms and help identify individuals at risk for developing chronic pain. This topic highlights an active area of research, with increasing efforts to characterize interindividual variability in CPM. Studies have examined the relationship between CPM capacity and brain correlates such as peak alpha frequency [25] and functional brain connectivity patterns in healthy individuals [26, 27] and patients with central neuropathic pain [28]. Although the intensity of the conditioning stimulus in CPM paradigms might appear to be an obvious determinant of CPM capacity, prior findings have not consistently supported this assumption. Importantly, it has been suggested that a minimal conditioning pain intensity is necessary to effectively engage the descending inhibitory mechanisms underlying CPM [29]. However, several studies have reported no clear association between conditioning stimulus intensity and the magnitude of CPM capacity [29–31]. These observations suggest that subjective pain ratings alone are poor predictors, and that more objective, physiological factors may better account for interindividual variability in endogenous pain modulation.

In this regard, cardiovascular measures may provide physiological insights, given the extensively investigated, yet still incompletely understood, bidirectional interplay between the autonomic and nociceptive systems mediated by shared neuronal structures [32–34]. Notably, greater CPM capacity has been associated with higher heart rate variability (HRV) [35] and more pronounced blood pressure responses during the cold pressor test (CPT), a common conditioning stimulus in CPM paradigms [36–38]. Noxious cold input during the CPT elicits a coordinated sympathetic activation and an increase in blood pressure [39, 40]. This pressor response subsequently activates baroreceptors, which can elicit a decrease in heart rate (HR) and may engage descending pain inhibitory pathways, thereby contributing to the restoration of physiological homeostasis [34]. Consistent with these mechanisms, stronger baroreflex function, commonly measured via baroreflex sensitivity (BRS), has been linked to lower pain sensitivity [41, 42]. Moreover, greater OA capacity has been observed in individuals with lower resting HR [43] and higher HRV [44]. It is important to recognize that both endogenous pain modulation and cardiovascular parameters are inherently variable and influenced by multiple factors. In particular, psychological characteristics, including higher pain catastrophizing [45], depression [46], and anxiety scores [29, 45], have been associated with diminished endogenous pain modulatory capacity [47], whereas cardiovascular function is well known to be influenced by sex, age, and body mass index (BMI) [35, 48–50].

Overall, these findings suggest that cardiovascular adaptability may contribute to endogenous pain modulation and the maintenance of physiological homeostasis. Hence, this study aimed to examine the role of cardiovascular function in endogenous pain modulation by assessing a range of cardiovascular parameters related to both CPM and OA. Based on previous evidence, we hypothesized that participants exhibiting greater cardiovascular adaptability (i.e., higher resting HRV and BRS, greater blood pressure reactivity to stress) would exhibit enhanced endogenous pain modulatory capacity, reflected by stronger CPM and OA.

## 2. Methods

### 2.1. Participants

A total of 30 healthy, pain‐free participants aged 18–70 years were recruited for this study. This sample size was informed by previous studies examining the relationship between endogenous pain modulation and cardiovascular function [36, 51] and confirmed by a retrospective sensitivity analysis. Participants were excluded if they met any of the following criteria: (1) any medical condition affecting the peripheral or central nervous system (e.g., neurological or psychological disorders, systemic diseases predisposing to small‐fiber neuropathy such as diabetes), (2) the presence of acute or chronic pain (e.g., headaches, injury‐related pain), (3) cardiovascular disease, (4) pregnancy, or (5) regular medication intake other than birth control. Participants were instructed to abstain from nonprescription analgesics, alcohol, and vigorous‐intensity physical activity for 24 h and from nicotine and caffeine for at least 4 h prior to their scheduled visit.

### 2.2. Study Design

This study utilizes data from a larger multimodal protocol conducted during a single experimental visit. Prior to the visit, all participants completed psychological and pain questionnaires independently. The experimental procedures were then conducted in the following order: a brief sensory evaluation to confirm intact somatosensory function, followed by multimodal pain habituation paradigms (including electrophysiological measures such as contact heat‐evoked potentials), quantitative sensory testing, a temporal summation of pain paradigm, and the OA paradigm. Following this, cardiovascular recordings were initiated, including measurements during the Valsalva maneuver and continuous monitoring throughout a 5‐min baseline period, a 2‐min CPT, and a 2‐min post‐CPT recovery period. Prior to baseline recording, participants rested quietly for approximately 5 min. The CPT simultaneously served as the conditioning stimulus for the CPM paradigm. However, the present study focused solely on cardiovascular assessments and measures of endogenous pain modulation (i.e., OA, CPM) as depicted in Figure 1.

**FIGURE 1:**
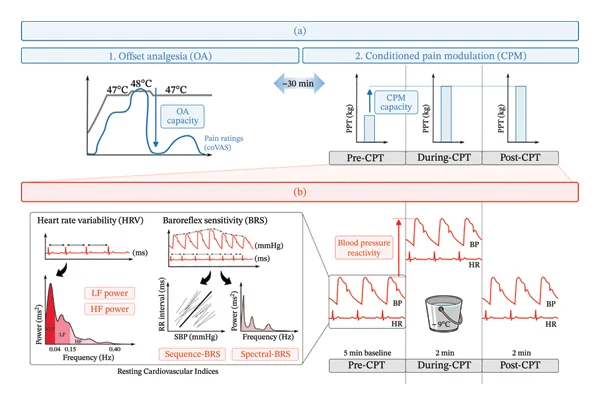
Experimental setup. (a) Endogenous pain modulation was assessed in the following order: OA, followed by CPM using parallel and sequential designs, separated by approximately 30 min. Primary outcome measures reflecting endogenous pain modulatory capacity are indicated in blue boxes. (b) Cardiovascular function was assessed using continuous noninvasive blood pressure and electrocardiographic recordings obtained during a 5‐min baseline period, a 2‐min during‐CPT period, and 2‐min post‐CPT recovery period. Resting cardiovascular indices, including heart rate variability and baroreflex sensitivity, were derived from signal processing of the 5‐min baseline recording. Blood pressure reactivity was quantified as the relative change from baseline to the CPT period. Primary cardiovascular outcome measures are highlighted in red boxes. Abbreviations: BP; blood pressure; coVAS, computerized visual analog scale; CPT, cold pressor test; HR, heart rate; HF, high frequency, LF, low frequency; PPT, pressure pain threshold.

All assessments were conducted in a quiet, temperature‐controlled room (∼22°C), and were obtained with participants in a seated position. All procedures conducted during the study were in accordance with the Declaration of Helsinki, and all participants provided written informed consent before commencement of the study. The study received approval from the local ethics committee “Kantonale Ethikkommission Zürich, KEK” (ref. number: 2024‐00134).

### 2.3. Questionnaires and Sensory Evaluation

Prior to the visit, participants were asked to fill out electronic versions of the Pain Catastrophizing Scale (PCS) [52] and the Hospital Anxiety Depression Scale (HADS) [53] to assess potential confounding effects of pain catastrophizing, anxiety, and depression on pain experience. At the beginning of the visit, sensory bedside testing was conducted to confirm intact somatosensory function in the designated test area (right hand) as a prescreening procedure, ensuring that exclusion Criterion 1 (e.g., polyneuropathy) was not present. Assessment included light touch, sharp‐dull discrimination with a safety pin, vibration sensation (64‐Hz Rydel‐Seiffer tuning fork), and thermal perception using 25°C and 40°C thermorollers (Somedic, Hörby, Sweden). Intact light touch was defined as correct perception of ≥ 8/10 stimuli, referenced to the facial region. Sharp‐dull discrimination was assessed using 10 stimulus pairs, with normal function defined as ≥ 8/10 correct pairs. Vibration sensation was considered intact if scores exceeded 6/8 on the Rydel‐Seiffer scale. For thermal testing, normal function was defined as ≥ 8/10 correct identifications of warm and cold stimuli. Participants exhibiting pathological findings in any of the above assessments were not included in the study.

### 2.4. Endogenous Pain Modulation

The CPM protocol employed pressure pain thresholds (PPTs) as test stimuli and immersion of the right hand in a cold‐water bath (9°C ± 0.5°C) for 2 minutes as the conditioning stimulus. In line with recommendations that the conditioning stimulus should be sufficiently painful [29], but tolerable, fixed cold‐water temperatures around 10°C represent a practical compromise between tolerability and effective CPM induction [54–56]. This combination was selected based on prior literature, indicating that PPT assessed via pressure algometry, paired with cold‐water immersion, has been found to yield robust and reliable CPM effects [57]. PPTs were measured at the left deltoid muscle using an analog pressure algometer (FDN100, Wagner Instruments) equipped with a circular silicone rubber tip (1 cm diameter). Thresholds were determined using the method of limits, with pressure increasing at a rate of 0.5 kg/s. PPTs were assessed at three time points: prior to, during, and immediately following the cold‐water bath. During hand immersion in the cold‐water bath, participants rated the perceived pain intensity every 30 s using a numeric rating scale (NRS), and the average of these ratings was calculated. Finally, the CPM capacity was determined by calculating the percentage change in PPTs during (parallel CPM design) or after the noxious conditioning stimulus (sequential CPM design) relative to baseline. Negative CPM values indicate pain inhibition, whereas positive values reflect pain facilitation.

OA was assessed using the standardized three‐phase thermal paradigm originally described by Grill and Coghill [13]. Thermal stimuli were delivered via a computer‐controlled contact heat stimulator (Pathway, Medoc Advanced Medical Systems, Ramat Yishai, Israel), with an advanced thermal stimulator (ATS) thermode (30 × 30 mm contact surface) positioned on the volar surface of the nondominant forearm. The paradigm comprised three consecutive thermal phases, initiated from a baseline temperature of 35°C. The temperature first increased at a rate of 1.5°C/s to 47°C and was maintained for 5 s (T1). It was then elevated by 1°C–48°C at the same ramp rate and sustained for an additional 5 s at 48°C (T2). Subsequently, the temperature was decreased to 47°C and held constant for 20 s (T3), before returning to baseline at a cooling rate of 6°C/s. Throughout the heat stimulation sequence, participants provided continuous pain ratings via a computerized visual analog scale (coVAS, Medoc Advanced Medical Systems, Ramat Yishai, Israel), which ranged from “*no pain*” (left anchor) to “*worst imaginable pain*” (right anchor), allowing for real‐time, continuous quantification of perceived pain intensity. To quantify OA, the maximum pain rating during the second phase (T2_max_) and the minimum pain rating during the third phase (T3_min_) were identified for each participant. The slope between T2_max_ and T3_min_ was computed to quantify the rate of decline in pain ratings during the rapid inhibitory effect. OA capacity was expressed as the percentage decrease in pain intensity from T2_max_ to T3_min_. More negative OA capacity values reflect stronger pain inhibition. An overview of all measures used to assess endogenous pain modulation is provided in Figure 1a.

### 2.5. Cardiovascular Assessments

An overview of all cardiovascular outcome measures is provided in Figure 1b. Participants rested quietly for 5 min while seated. Afterward, continuous noninvasive blood pressure measurements, including systolic blood pressure (SBP), diastolic blood pressure (DBP), and mean arterial pressure (MAP), were obtained using a hemodynamic monitoring system (Finapres NOVA, Finapres Medical Systems, Amsterdam, Netherlands). A size‐appropriate finger cuff was positioned on the mid‐phalanx of the left middle finger, and hydrostatic pressure differences between finger and heart level were continuously corrected using the system’s heart reference unit. Additionally, electrocardiographic (ECG) activity was recorded using a standard three‐lead configuration with disposable Ag/AgCl electrodes (Kendall H124SG, Cardinal Health, Germany). Prior to electrode placement, the skin was cleaned with alcohol (Softasept N, B. Braun Medical AG, Germany) to minimize impedance. Electrodes were positioned below the right and left clavicles and along the left lower rib cage. ECG signals were band‐pass filtered between 0.3 and 1000 Hz and sampled at 1000 Hz using the PowerLab data acquisition system (Dual Bio Amp; ADInstruments, Dunedin, New Zealand).

Continuous cardiovascular recordings were obtained across three experimental phases: a 5‐min baseline preceding the CPT (pre‐CPT), a 2‐min CPT‐induced stress phase (during‐CPT), and a 2‐min recovery phase (post‐CPT). Data were processed offline using LabChart software (ADInstruments, Dunedin, New Zealand). Blood pressure reactivity to the CPT (including SBP, DBP, and MAP) was quantified as the percentage change from baseline to the peak blood pressure value observed during immersion. Resting cardiovascular signals acquired during the baseline period were further analyzed to derive resting HRV and BRS.

HRV analysis was performed using the LabChart HRV module (Version 2.0.3) [58, 59]. ECG artefacts (e.g., ectopic beats, arrhythmic events, or noise) were identified and removed following visual inspection by two experienced ECG readers, with R–R intervals constrained to 600–1400 ms and complexity parameters set between 0.6 and 1.5. The present analyses focused on frequency‐domain HRV metrics to reduce data complexity and facilitate a clearer differentiation of underlying autonomic mechanisms. Thus, frequency‐domain analysis was conducted using the Lomb–Scargle periodogram, a spectral estimation method well suited for unevenly sampled data such as R–R interval time series, thereby avoiding the need for interpolation or resampling and preserving the integrity of the original data. Power spectral density was extracted for the low‐frequency (LF; 0.04–0.15 Hz) and high‐frequency (HF; 0.15−0.4 Hz) bands and normalized to total power. These measures were selected because they are commonly used HRV metrics [60, 61]. Under resting seated conditions, HRV‐LF reflects cardiac autonomic modulation involving vagal and baroreflex‐related contributions and should not be interpreted as a specific index of cardiac sympathetic activity [62, 63]. HRV‐HF power reflects predominantly parasympathetically mediated HR regulation [60]. Nevertheless, time‐domain HRV metrics were also derived, namely, root mean square of successive R–R interval differences (RMSSD) and standard deviation of all normal R–R intervals (SDRR), as these parameters are more widely implemented in commercially available devices.

BRS was assessed from spontaneous beat‐to‐beat fluctuations in SBP and HR, providing a noninvasive measure of baroreflex function [64]. Continuous beat‐to‐beat SBP and simultaneous ECG signals were analyzed during the baseline period as described above. BRS was quantified using both time‐domain (sequence‐BRS) [65] and frequency‐domain (spectral‐BRS) [66, 67] approaches using a custom script developed in R (Version 4.5.2, R Foundation for Statistical Computing, Vienna, Austria). Spontaneous baroreflex sequences were defined as ≥ 3 consecutive beats with SBP changes ≥ 1 mmHg accompanied by concordant R–R interval changes ≥ 1 ms and a correlation coefficient *r* ≥ 0.85 [65, 68]. Sequence‐BRS was quantified as the slope of the linear regression between SBP and corresponding R–R interval within each valid sequence and expressed as the mean slope (ms/mmHg) across baseline. Spectral‐BRS was derived from the transfer function between SBP and R–R interval variability within the LF band (0.04–0.15 Hz), with segments exhibiting coherence ≥ 0.5 noted as a metric of signal quality rather than a strict exclusion criterion for primary analyses. The LF range was selected because it reflects spontaneous oscillations strongly modulated by sympathetic and parasympathetic interactions and Mayer wave dynamics [66]. Spectral‐BRS in this frequency range reflects baroreflex gain across continuous oscillatory components of SBP and R–R variability, thereby capturing the dynamic coupling between cardiovascular signals over a defined frequency spectrum [69, 70]. The sequence method identifies discrete, spontaneous beat‐to‐beat sequences of concordant SBP and R–R interval changes and predominantly reflects parasympathetic modulation, with limited sensitivity to slower, sympathetically mediated oscillations [71]. For this reason, we used a combination of both approaches, as they provide complementary physiological information.

### 2.6. Statistical Analyses

All statistical analyses were performed using R statistical software (Version 4.5.2, R Foundation for Statistical Computing, Vienna, Austria). Normal distribution was tested using Shapiro–Wilk tests and complemented by visual inspections of histograms and quantile–quantile plots. A significance level of *α* = 0.05 was employed.

To evaluate changes in SBP, DBP, and MAP across CPT timepoints (i.e., pre‐, during‐, and post‐CPT), linear mixed‐effects models fitted with the lmer() function from the lme4 package in R were used. In each model, the respective cardiovascular parameter served as the dependent variable, timepoint was included as a fixed effect, and participant ID was specified as a random intercept to account for interindividual variability. Normality of model residuals was assessed using histograms, residuals‐versus‐fitted plots, and quantile–quantile plots, with Shapiro–Wilk tests used as a supplementary diagnostic. Post hoc pairwise comparisons between timepoints were conducted with Bonferroni adjustments to control for multiple testing.

To assess whether CPM and OA induced pronounced pain inhibition significantly different from zero, separate one‐sample Wilcoxon signed‐rank tests were performed for each paradigm.

Relationships between cardiovascular (i.e., resting HRV/BRS and blood pressure reactivity) and endogenous pain modulatory parameters (i.e., OA and CPM) were examined using either Pearson or Spearman rank correlations, selected based on the distributional properties of the data. To account for multiple comparisons, *p* values were adjusted using the Benjamini–Hochberg correction, applied separately to each primary CPM and OA analysis (five hypotheses each). Where needed, partial correlations were performed to account for confounding effects. Potential confounding effects on cardiovascular parameters were examined as follows: Sex differences were assessed using independent‐samples *t*‐tests or Mann–Whitney *U*‐tests, as appropriate, and associations with age and BMI were evaluated using Pearson’s or Spearman’s rank correlations, depending on data distribution. Additionally, baseline SBP was included as a covariate in the partial correlation analyses for BRS, given its well‐established influence on BRS, particularly in older adults, who were represented in the present sample [72, 73]. Moreover, circadian variation represents a potential confounding factor, as testing sessions could not always be scheduled at the same time of day. Autonomic tone exhibits well‐established diurnal fluctuations, including a shift toward sympathetic predominance during the morning transition from sleep to wakefulness, coinciding with peak cortisol levels [74]. Likewise, HR increases upon awakening, reaches a peak between 10:00 and 12:00 h, and gradually declines thereafter [75, 76]. To simplify the influence of circadian rhythms, participants were categorized into early and late testing groups. A cutoff at 13:00 h was selected as a pragmatic approach that yielded two similarly sized groups while broadly capturing morning versus afternoon testing sessions. Group differences in autonomic measures were assessed using independent‐samples *t*‐tests or Mann–Whitney *U*‐tests, as appropriate.

Furthermore, relationships between psychological variables (i.e., PCS and HADS) and endogenous pain modulation (i.e., CPM and OA) were analyzed using correlation analyses to identify potential psychological confounders.

## 3. Results

### 3.1. Demographic and Psychological Characteristics

No participants exhibited any sensory abnormalities during the bedside testing; therefore, 30 healthy participants were included in the study. Participant demographics and self‐reported questionnaire results are presented in Table 1. Consistent with expectations for a healthy population, none of the participants exhibited clinically relevant scores for the PCS (≥ 30 points) [77] or for either the depression or anxiety subscales of the HADS (≥ 8 points for each) [53]. The higher proportion of male participants in this cohort reflects its intended use as a control group in a forthcoming study involving a clinical cohort with spinal cord injury‐related neuropathic pain, which has a predominance of male patients [78].

**TABLE 1:** Participant characteristics and psychological functioning.

Characteristic	Healthy participants (*N* = 30)
Sex (M/F)	21/9
Age (years)	48.2 ± 12.7
BMI (kg/m^2^)	24.1 ± 2.5
PCS (score, range: 0–52)	4.6 ± 5.5
HADS depression (score, range: 0–21)	0.8 ± 1.4
HADS anxiety (score, range: 0–21)	1.8 ± 1.7

*Note:* Values are reported as mean and standard deviation.

Abbreviations: BMI, body mass index; HADS, Hospital Anxiety and Depression Scale; PCS, Pain Catastrophizing Scale.

### 3.2. Resting Cardiovascular Indices

Continuous cardiovascular recordings could not be obtained for one participant because anatomical features of the finger prevented adequate placement of the finger cuff. In an additional participant, sequence‐BRS could not be calculated because fewer than three consecutive valid sequences with concordant increases or decreases in SBP and R–R interval were identified during the 5‐min baseline.

The following baseline HRV parameters were measured: HRV‐LF power 38.1% ± 18.5% and HRV‐HF power 20.8% ± 14.6% (Figure 2a). Resting BRS averaged 7.7 ± 3.5 ms/mmHg for time‐domain (sequence‐BRS) and 8.6 ± 4.0 ms/mmHg for frequency‐domain (spectral‐BRS) estimates. For spectral‐BRS, coherence values were below 0.5 in 15 participants, with these cases indicated by squares (Figure 2b).

**FIGURE 2:**
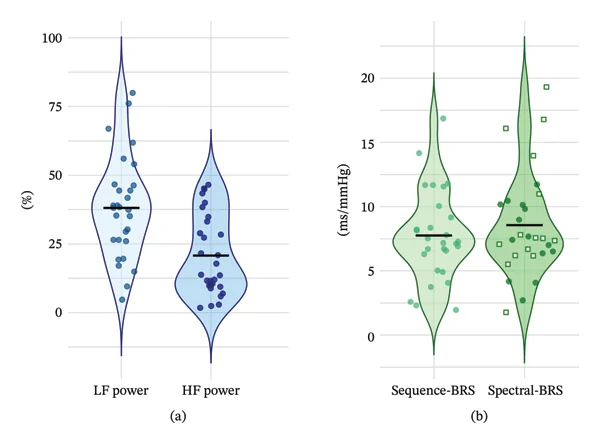
Resting cardiovascular indices. Violin plots illustrate the distribution of resting cardiovascular parameters with individual datapoints representing single participants; horizontal bars indicate group means. (a) Resting heart rate variability measures, including low‐frequency power and high‐frequency power. (b) Resting baroreflex sensitivity assessed in the time‐domain (sequence‐BRS) and frequency‐domain (spectral‐BRS). Squared datapoints indicate spectral‐BRS values with a coherence < 0.5. Abbreviations: BRS, baroreflex sensitivity; HF, high frequency; HRV, heart rate variability; LF, low frequency.

Examination of known influences such as age, sex, and BMI, on cardiovascular function, revealed several confounding effects within the dataset (see Supporting Table S1 for details). Specifically, males exhibited higher HRV‐LF power (41.3% ± 19.9%) compared with females (29.7% ± 11.2%, *p* = 0.031), whereas females showed higher HRV‐HF power (31.7% ± 16.0%) than males (16.6% ± 11.9%, *p* = 0.018), consistent with previous reports [79]. Furthermore, spectral‐BRS was higher in females (10.9 ± 4.0 ms/mmHg) compared to males (7.6 ± 3.8 ms/mmHg, *p* = 0.016) in the present dataset. In addition, the potential influence of testing time on cardiovascular function was examined, as it was not feasible to schedule all participants at the same time of day. No differences in cardiovascular function were observed between participants tested earlier versus later in the day, as shown in Supporting Table S2.

### 3.3. Blood Pressure Reactivity During CPT

Table 2 summarizes different blood pressure measures assessed at baseline, during‐, and post‐CPT, and blood pressure reactivity.

**TABLE 2:** Blood pressure parameters at rest (baseline), during, and after the CPT.

Parameter	Baseline (pre‐CPT)	During‐CPT	Post‐CPT	Reactivity (%)
SBP (mmHg)	128 ± 16	171 ± 22^∗^	133 ± 15	35 ± 20
DBP (mmHg)	80 ± 9	107 ± 12^∗^	82 ± 8	34 ± 18
MAP (mmHg)	96 ± 11	127 ± 14^∗^	99 ± 10	33 ± 18

*Note:* Values are reported as mean and standard deviation.

Abbreviations: CPT, cold pressor test; DBP, diastolic blood pressure; MAP, mean arterial pressure; SBP, systolic blood pressure.

^∗^ *p* < 0.001 for differences from baseline.

The CPT elicited significant increases in SBP, DBP, and MAP (Ts > 14.97, *p* < 0.001) compared to baseline. There was no significant difference between pre‐ and post‐CPT values for any blood pressure parameter (SBP, DBP, and MAP; all *p* > 0.05), suggesting a return to baseline levels after the CPT. No significant confounding effects of sex, age, or BMI were detected on any blood pressure parameter in the resting or reactive state (all *p* > 0.05; Supporting Table S1).

### 3.4. CPM and Associations With Cardiovascular Indices

Hand immersion in the cold‐water bath for 2 min elicited a mean pain intensity of 5.0 ± 2.2 (range 1.0–9.6), which all participants tolerated without withdrawing their hand. Mean PPT before the CPT was 5.2 ± 1.8 kg. During the CPT, mean PPT increased to 6.1 ± 2.2 kg (*T* = 4.57, *p* < 0.001), resulting in a pronounced inhibitory CPM capacity in the parallel CPM design (−19.0% ± 14.7%, *t* = −7, *p* < 0.001). Similarly, mean PPT increased to 6.1 ± 2.3 kg after the CPT (*T* = 4.16, *p* < 0.001), yielding a pronounced inhibitory CPM capacity in the sequential CPM design (−18.7% ± 23.7%, V = 37, *p* < 0.001). Psychological factors, including anxiety, depression, and pain catastrophizing, were not significantly associated with CPM capacity (all*p* > 0.05; Supporting Table S3).

Next, the relationship between cardiovascular function (i.e., HRV‐LF power, HRV‐HF power, sequence‐BRS, spectral‐BRS, and SBP reactivity) and parallel CPM capacity was examined. The present study focused on SBP because it shows greater sensitivity to short‐term autonomic fluctuations than DBP [80] or MAP [69] and is a well‐established clinical indicator of cardiovascular disease [81]. Given that sex was identified as a potential confounder of HRV‐LF power, HRV‐HF power, and spectral‐BRS, partial correlation analyses were performed to control for its influence. No statistically significant associations were observed between parallel CPM capacity and frequency‐domain HRV parameters (HRV‐LF power, HRV‐HF power) or sequence‐BRS (all *p* > 0.05; Figure 3a–c). In contrast, higher spectral‐BRS was significantly related to greater parallel CPM capacity, an association that remained significant after adjustment for sex and baseline SBP (rho = −0.52, *p* = 0.021, multiple‐testing corrected; Figure 3d). Furthermore, greater SBP increases during the CPT (i.e., SBP reactivity) were associated with greater parallel CPM capacity (rho = −0.48, *p*=0.021, multiple‐testing corrected; Figure 3e). Detailed results regarding the relationship between cardiovascular function and sequential CPM capacity are presented in Supporting Figure S1. Notably, greater SBP reactivity was likewise associated with higher sequential CPM capacity (rho = −0.49, *p* = 0.033; Supporting Figure S1e). Associations between time‐domain HRV parameters and parallel CPM capacity are shown in Supporting Figures S2a and S2b.

**FIGURE 3:**
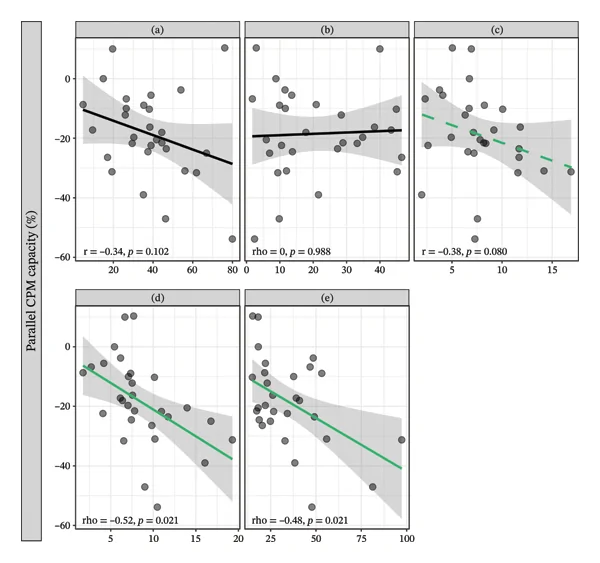
Partial correlation analyses between parallel CPM capacity and heart rate variability, baroreflex sensitivity, and blood pressure reactivity. Negative values for CPM capacity depict pain inhibition, while positive values show pain facilitation. Significant correlations are illustrated by a green regression line and nonsignificant correlations by a black regression line. Dashed green regression lines indicate significant correlations prior to multiple comparison correction. Partial correlations between CPM capacity and (a) HRV‐LF power adjusted for sex, (b) HRV‐HF power adjusted for sex, (c) sequence‐BRS adjusted for baseline SBP, (d) spectral‐BRS adjusted for baseline SBP and sex, and (e) SBP reactivity. *p* values were corrected using a Benjamini–Hochberg correction for a family of five tests. Abbreviations: BRS, baroreflex sensitivity; CPM, conditioned pain modulation; HF, high frequency; HRV, heart rate variability; LF, low frequency; SBP, systolic blood pressure.

Uncorrected and multiple‐comparison‐corrected *p* values for all correlations between parallel CPM capacity and cardiovascular parameters are reported in Supporting Table S4.

In a sensitivity analysis excluding spectral‐BRS estimates with a coherence < 0.5, the association between parallel CPM and spectral‐BRS maintained a consistent direction and magnitude (rho = −0.48). However, the reduced sample size yielded a wider confidence interval that did not reach statistical significance (*p* = 0.084).

### 3.5. OA and Associations With Cardiovascular Indices

All 30 participants tolerated and completed the three‐phase OA paradigm. Figure 4a illustrates individual coVAS pain ratings alongside the average, highlighting the characteristic sudden decrease in pain rating, i.e., the OA capacity. The mean pain rating at T2_max_ was 79.4 ± 24.0, and 12.0 ± 19.6 at T3_min_. On average, T2_max_ occurred 2.7 ± 1.6 s after the onset of 48°C, and T3_min_ was reached 5.9 ± 3.6 s after the onset of the second 47°C. The average rate of decline in pain intensity between T2_max_ and T3_min_ was −12.0 ± 8.0 coVAS/s, reflecting the characteristic sudden and fast OA effect. The mean OA capacity was −85.7% ± 21.8%, reflecting a pronounced pain inhibitory effect (*V* = 0, *p* < 0.001; Figure 4b). OA capacity was not associated with psychological factors such as anxiety, depression, or pain catastrophizing (*p* > 0.05; see Supporting Table S3 for details).

**FIGURE 4:**
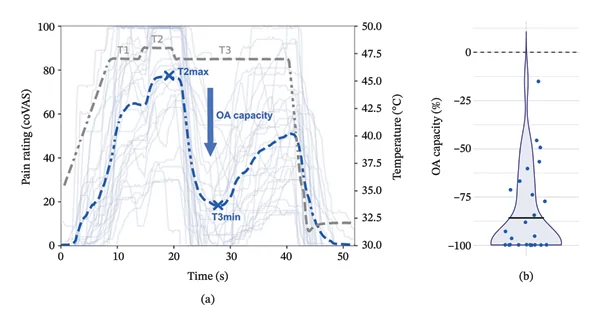
Average heat pain ratings during the OA paradigm and OA capacity. (a) Schematic of the thermal stimulation protocol (T1–T2–T3; 47°C–48°C–47°C; dashed gray line), individual pain ratings (light blue lines), and grand average across all participants (dark blue line). Markers indicate the averaged timepoints T2_max_, T3_min_, and the resulting OA capacity, all highlighted in dark blue. (b) Violin plot illustrates the distribution of OA capacity, calculated as the percentage reduction from T2_max_ to T3_min_. Individual datapoints representing single participants and the horizontal bar indicates the group average. Abbreviations: coVAS, computerized visual analog scale; OA, offset analgesia.

Furthermore, associations between cardiovascular function (i.e., HRV‐LF power, HRV‐HF power, sequence‐BRS, spectral‐BRS, and SBP reactivity) and OA capacity were examined. Among frequency‐domain HRV measures, only HRV‐LF power revealed a significant correlation with OA capacity, which remained significant after adjustment for sex (rho = −0.50, *p* = 0.030, multiple‐testing corrected; Figure 5a). In addition, higher spectral‐BRS was significantly related to greater OA capacity, persisting after adjustment for sex and baseline SBP (rho = −0.48, *p* = 0.030, multiple‐testing corrected; Figure 5d). Substantiating this relationship, a subanalysis demonstrated that higher spectral‐BRS was associated with a faster rate of decline for OA (rho = −0.40, *p* = 0.035). No significant associations were observed between OA capacity and HRV‐HF power (Figure 5b), sequence‐BRS (Figure 5c), or SBP reactivity (Figure 5e; all *p* > 0.05). Associations between time‐domain HRV parameters and OA capacity are depicted in Supporting Figures S2c and S2d. Finally, OA capacity was not associated with either parallel CPM capacity (rho = 0.04, *p* = 0.856) or sequential CPM capacity (rho = −0.17, *p* = 0.375), supporting the complementary value of using both paradigms for a more comprehensive characterization of endogenous pain modulation.

**FIGURE 5:**
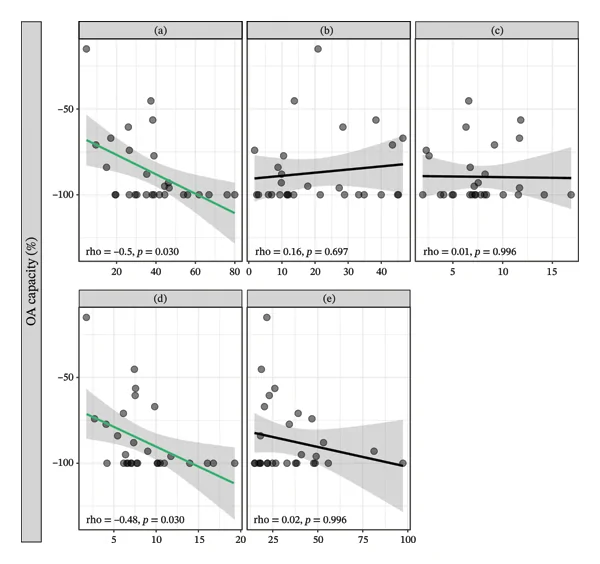
Partial correlation analyses between OA capacity and heart rate variability, baroreflex sensitivity, and blood pressure reactivity. Negative values for OA capacity depict pain inhibition. Significant correlations are illustrated by a green regression line and nonsignificant correlations by a black regression line. Partial correlations between OA capacity and (a) HRV‐LF power adjusted for sex, (b) HRV‐HF power adjusted for sex, (c) sequence‐BRS adjusted for baseline SBP, (d) spectral‐BRS adjusted for baseline SBP and sex, and (e) SBP reactivity. *p* values were corrected using a Benjamini–Hochberg correction for a family of five tests. Abbreviations: BRS, baroreflex sensitivity; CPM, conditioned pain modulation; HF, high frequency; HRV, heart rate variability; LF, low frequency; OA, offset analgesia; SBP, systolic blood pressure.

Uncorrected and multiple‐comparison‐corrected *p* values for all correlations between OA capacity and cardiovascular parameters are reported in Supporting Table S4.

## 4. Discussion

The present study aimed to investigate the contribution of cardiovascular function to endogenous pain modulation by assessing various cardiovascular parameters in relation to both CPM and OA. Importantly, higher baroreflex function during rest was related to both greater CPM and OA capacity. Additionally, more pronounced blood pressure responses to the CPT were associated with greater CPM capacity, demonstrating for the first time a robust relationship between cardiovascular reactivity and endogenous pain modulation. These findings strongly suggest that cardiovascular adaptability is closely linked to the efficiency of endogenous pain modulatory processes. Given that reduced cardiovascular adaptability [82, 83] and impaired pain modulation [4, 7, 8] are frequently observed in chronic pain conditions, these results may have important implications for understanding the pathophysiology of chronic pain. The potential mechanisms underlying the coupling between cardiovascular function and endogenous pain modulation are addressed in the following sections.

### 4.1. Blood Pressure Reactivity During the CPT and Its Association With CPM

The present findings demonstrate that greater blood pressure reactivity to the CPT is moderately associated with higher CPM capacity, observed for the first time across both parallel and sequential CPM designs. Our results align with prior studies reporting the relationship between CPM capacity and blood pressure responses in sequential designs when the CPT was applied as a conditioning stimulus [36–38]. In contrast, a study employing ischemic pain as a conditioning stimulus failed to observe this relationship, suggesting that it may depend on the type of conditioning stimulus employed. Notably, ischemic pain elicited only small blood pressure increases and relatively weak CPM effects [84]. Interestingly, a study by Streff et al. comparing different conditioning stimuli (interdigital web pinching versus hot water immersion) reported comparable perceived pain intensities across modalities. However, hot water immersion induced greater cardiovascular stress reactivity and elicited stronger CPM effects than the web pinching technique [85]. These findings suggest that cardiovascular stress reactivity during conditioning, rather than pain intensity per se, may contribute to effective descending pain inhibition.

### 4.2. The Role of Baroreflex‐Mediated Mechanisms in Endogenous Pain Modulation

According to established criteria, normal baroreflex function is defined as greater than 6 ms/mmHg, moderate baroreflex dysfunction as between 3 and 6 ms/mmHg, and severe baroreflex dysfunction as less than 3 ms/mmHg [67, 86, 87]. In the present data, three participants demonstrated low sequence‐BRS values, and two had low spectral‐BRS values. It is well established that baroreceptor activation is associated with reduced pain sensitivity, a phenomenon often referred to as baroreflex‐mediated analgesia [32, 42, 88]. Moreover, a positive moderate relationship between baroreflex function during CPT and CPM capacity was reported [89]. Notably, this association was observed exclusively when an active baroreceptor stimulation protocol was applied during the CPM paradigm. Extending this body of evidence, we demonstrated that resting BRS is strongly associated with CPM capacity in a healthy population, even in the absence of experimental baroreceptor modulation. Our results are consistent with the assumption that baroreflex‐mediated mechanisms may trigger descending pain inhibition [90, 91], and consequently reduce pain sensitivity. However, this association did not remain significant for the sequence‐derived BRS measure after correction for multiple comparisons. The stronger relationship between spectral‐derived BRS and CPM capacity, compared with sequence‐derived BRS, may be attributable to underlying physiological and methodological differences between the two assessment techniques. Spectral‐derived BRS captures spontaneous SBP–R–R interval coupling within the LF band, where a coherence threshold (> 0.5) is traditionally applied to guarantee reliable gain estimation [92, 93]. However, the use of an arbitrary coherence criterion has recently been criticized because coherence values tend to decrease with impaired baroreflex function, potentially resulting in systematic exclusion of individuals with low BRS [67]. In the present sample, strict application of this criterion would have resulted in the exclusion of nearly half of the recordings, leading to substantial data loss and limited statistical power. Therefore, consistent with newer recommendations, transfer function gain was averaged across the entire LF band irrespective of coherence values [94, 95]. This approach enhances robustness by integrating baroreflex gain over the full frequency range, at the cost of reduced sensitivity to coherence fluctuations, which could mask the underlying relationship with pain modulation. However, a sensitivity analysis suggests that this risk is minimal, as excluding low‐coherence values preserved the magnitude and direction of the observed effect. Therefore, spectral‐BRS may reflect baroreflex‐related cardiovascular regulation in a manner that differs from sequence‐based estimates [69, 70] and may provide a measure that is more relevant to endogenous pain modulation. Sequence‐ and spectral‐BRS quantify different features of spontaneous SBP–R–R interval coupling. Sequence‐BRS is derived from brief beat‐to‐beat runs of concordant changes, whereas spectral‐BRS evaluates slower continuous oscillatory coupling across the entire recording and may therefore capture a broader range of baroreflex‐related cardiovascular dynamics. The stronger association observed for spectral‐BRS may therefore reflect physiological differences and greater use of the available data. Additionally, the sequence technique predominantly reflects parasympathetic BRS and is less sensitive to slower sympathetic‐driven oscillations [67, 71]. Differences in measurement reliability, particularly the limited number of spontaneous sequences detected for the analysis, may have attenuated associations in the time‐domain approach. In line with this interpretation, Chalaye and colleagues did not observe a relationship between sequence‐derived BRS at rest and CPM capacity [36], possibly due to reliance on time‐domain methodology, which may insufficiently capture sympathetically mediated processes in baroreflex function.

The relationship between baroreflex function and descending pain inhibition may be explained by physiological links between cardiovascular regulation and endogenous pain modulatory systems [90, 91]. The nucleus tractus solitarius is a key interface between autonomic and sensory nervous systems. It is the primary site of synaptic input for the baroreflex and also constitutes a critical relay in descending pain inhibition [90]. Baroreceptors are mechanosensitive receptors located primarily in the aortic arch and carotid sinus. They respond to an arterial wall stretch from beat‐to‐beat fluctuations in systemic blood pressure through a global inhibitory effect on the sympathetic nervous system [96]. Through afferent signaling, they modulate sympathetic and parasympathetic outflow to the heart and peripheral vasculature, thereby regulating HR and vascular tone in response to changes in blood pressure [97], thus reflecting greater cardiovascular adaptability. Given that the CPT serves both as a conditioning stimulus in CPM paradigms [4] and as a strong sympathetic activator [98], an important consideration is the extent to which the inhibitory effect observed during CPM reflects a genuine nociceptive mechanism versus a baroreflex‐mediated analgesic response. CPT‐induced sympathetic activation acutely elevates arterial blood pressure, increasing baroreceptor firing and potentially engaging baroreflex‐mediated descending inhibitory pathways [99]. In this context, the reduction in pain perception observed during CPM may not solely represent a purely nociceptive mechanism but may also partly reflect baroreflex‐mediated analgesia. Thus, the inhibitory effect observed during CPM may arise from overlapping and superimposing mechanisms, encompassing both classical descending pain inhibition and a baroreceptor‐mediated mechanism.

Importantly, the present study builds on previous work by showing that resting baroreflex function is strongly associated with descending pain inhibition in CPM and, notably, also moderately associated in OA. To the best of our knowledge, this is the first study linking resting baroreflex function with OA. Unlike CPM, OA does not rely on CPT‐induced sympathetic activation and involves temporal filtering mechanisms of sensory information [13, 14]. Although the precise mechanisms underlying OA remain unclear, neuroimaging studies have identified activation of brain regions involved in pain modulation, including the brainstem, insula, and dorsolateral prefrontal cortex [11, 100]. In particular, OA has been linked to activation within the periaqueductal gray and the rostroventral medulla, key components of descending pain modulatory pathways that are also implicated in supraspinal regulation of cardiac function, such as HR regulation [100]. Experimental evidence indicated that pharmacologically induced reductions in HR via clonidine administration are associated with enhanced OA [43]. This suggests that increased parasympathetic function may support dynamic cardiovascular adjustments that are linked to more effective descending pain inhibition.

HRV captures the beat‐to‐beat variation driven by dynamic autonomic regulation. At rest, HRV is predominantly influenced by cardiac vagal activity [62], with higher HRV indicating more flexibility to adapt to changing demands [101]. In the present study, resting HRV values were largely within normative ranges reported for short‐term ECG recordings in healthy populations [102]. Given that endogenous pain modulation critically depends on a finely tuned interplay between facilitatory and inhibitory mechanisms, an association between HRV and endogenous pain modulation is conceptually plausible. Despite this theoretical overlap, empirical investigations directly examining the relationship between HRV at rest and endogenous pain modulation remain sparse.

One study reported that healthy individuals with higher HRV demonstrated a measurable OA effect, whereas those with lower HRV did not [44]. Our findings of a strong relationship between greater HRV‐LF power at rest and enhanced OA capacity are in line with this study. Because HRV‐LF power is affected by multiple autonomic and baroreflex‐related influences [62, 103], its association with OA could be seen as consistent with a relationship between cardiovascular autonomic regulation and endogenous pain modulation. However, in the absence of a constant‐temperature control condition, a potential contribution of habituation processes to the observed OA responses cannot be completely ruled out. Such processes may have contributed to the observed effects and could therefore have resulted in an overestimation of OA responses. In the context of CPM, prior work has demonstrated a significant, moderate correlation between pain inhibitory capacity and HRV‐LF power assessed during the CPT in healthy individuals [51]. Moreover, baseline time‐domain HRV measures have been reported to correlate with CPM responses in children and adolescents [104], as well as in healthy adult males [35]. In line with these findings, we found associations between higher baseline time‐domain HRV (both RMSSD and SDRR) and parallel CPM capacity in healthy individuals.

Collectively, the observed associations indicate that individuals exhibiting higher baroreflex function at rest, an index of autonomic adaptability, also demonstrate more effective activation of descending pain inhibitory pathways. This enhanced regulatory flexibility may facilitate a more rapid restoration of physiological homeostasis and underscores the integrative role of baroreflex‐mediated mechanisms as a neurophysiological link between cardiovascular regulation and endogenous pain modulation.

## 5. Conclusion

We provide evidence for a close interplay between cardiovascular regulation and endogenous pain modulation in healthy individuals. Greater BRS, a proxy for autonomic flexibility, was consistently associated with enhanced pain inhibitory capacity across both CPM and OA paradigms. These results suggest that baroreflex‐mediated mechanisms may represent a functional interface between autonomic control and descending pain inhibition. Given that both impaired baroreflex function [105] and deficient pain inhibition [4] are characteristic of chronic pain conditions, disrupted pain‐cardiovascular coupling may contribute to the development or maintenance of chronic pain. Elucidating the mechanisms underlying this coupling may help inform targeted interventions aimed at restoring autonomic flexibility and optimizing endogenous pain modulation. Nevertheless, these associations should be replicated in larger samples to confirm their robustness and generalizability.

## 6. Limitations

One limitation of the present study, as briefly discussed above, is the inclusion of all coherence values, which may reduce sensitivity to periods of stronger SBP–R–R interval coupling and potentially introduce additional variability in BRS estimates. Nevertheless, this approach allowed retention of data from participants with lower baroreflex function. The findings should therefore be interpreted with caution and replicated in larger samples using predefined signal quality criteria. Another methodological limitation concerns the absence of a constant‐temperature control condition in the OA paradigm, originally proposed by Grill and Coghill [13] to control for habituation or peripheral sensory adaptation during tonic heat stimulation. Although such a control condition may help isolate genuine OA effects, more recent evidence suggests that OA reflects mechanisms distinct from habituation processes [106]. Lastly, a further limitation is that lifestyle factors such as physical activity, fatigue, sleep quality, and nutritional status were not assessed, despite their known influence on cardiac autonomic nervous system activity [107] and pain perception [108, 109].

## Author Contributions

This study was designed by M.H. and J.K.M. Data were collected by J.K.M. and S.S. and analyzed by S.J.T.B. and J.K.M. Interpretation of the results was discussed by S.J.T.B., J.K.M., T.E.N., and M.H., with M.H. supervising the entire study. The manuscript was drafted by J.K.M.

## Funding

The study was funded by the Swiss National Science Foundation (SNSF), 32003B_200482; the Neuroscience Center Zurich (ZNZ); and the International Foundation for Research in Paraplegia (IRP), P202F.

## Disclosure

All authors reviewed and approved the final version.

## Conflicts of Interest

The authors declare no conflicts of interest.

## Supporting Information

Additional supporting information can be found online in the Supporting Information section.

## Supporting information


**Supporting Information 1** Table S1: Potential confounding effects of sex, age, and BMI on cardiovascular function. Table S2: Potential confounding effects of testing time on autonomic tone. Table S3: Bivariate correlation matrix between pain inhibitory capacity and psychological characteristics. Table S4: Uncorrected and corrected *p* values of all correlations.

**Supporting Information 2** Figure S1: Partial correlation analyses between sequential CPM capacity and heart rate variability, baroreflex sensitivity, and blood pressure reactivity.

**Supporting Information 3** Figure S2: Partial correlation analyses between endogenous pain modulation and time‐domain HRV parameters.

## Data Availability

The data that support the findings of this study are available from the corresponding author upon reasonable request.
