# Supplementary material for: The Role of Cardiovascular Function in Endogenous Pain Modulation
Source: Pain Res Manag. 2026 Nov 5;2026:8018460. doi: 10.1155/prm/8018460 (PMC13737465; doi:10.1155/prm/8018460)
Supplement: Supplementary file 1 [file PRM-2026-8018460-s002.docx]

# Supplementary

|  | **Sex** | **Age** | **BMI** |
| --- | --- | --- | --- |
| **Resting cardiovascular indices** | | | |
| HRV-LF power | M: 41.3 ± 19.9 %  F: 29.7 ± 11.2 %  **p=0.03** | -0.10 (0.59) | -0.11 (0.61) |
| HRV-HF power | M: 16.6 ± 11.9 %  F: 31.7 ± 16.0 %  **p=0.02** | -0.20 (0.30) | -0.19 (0.33) |
| Sequence-BRS | M: 7.1 ± 3.4 ms/mmHg  F: 9.5 ± 3.5 ms/mmHg  p=0.06 | -0.36 (0.06) | -0.28 (0.15) |
| Spectral-BRS | M: 7.6 ± 3.8 ms/mmHg  F: 10.9 ± 4.0 ms/mmHg  **p=0.02** | -0.30 (0.11) | -0.38 (0.06) |
| SBP | M: 129.5 ± 11.6 mmHg  F: 125.5 ± 23.8 mmHg  p=0.30 | 0.21 (0.26) | 0.21 (0.28) |
| DBP | M: 82.0 ± 7.0 mmHg  F: 75.5 ± 11.6 mmHg  p=0.14 | 0.21 (0.26) | 0.15 (0.42) |
| MAP | M: 97.8 ± 7.5 mmHg  F: 93.4 ± 16.7 mmHg  p=0.24 | 0.27 (0.14) | 0.13 (0.49) |
| **Cardiovascular reactivity** | | | |
| SBP reactivity | M: 33.9 ± 13.1 %  F: 37.5 ± 32.4 %  p=0.40 | -0.07 (0.72) | -0.03 (0.87) |
| DBP reactivity | M: 33.4 ± 12.7 %  F: 37.2 ± 27.6 %  p=0.68 | -0.19 (0.32) | -0.08 (0.69) |
| MAP reactivity | M: 32.2 ± 12.6 %  F: 36.4 ± 29.6 %  p=0.65 | -0.12 (0.55) | -0.07 (0.74) |

Table S1 – Potential confounding effects of sex, age and BMI on cardiovascular function. Sex differences were evaluated using independent t-tests or Mann-Whitney-U tests, as appropriate. Associations of age and BMI with blood pressure parameters were examined using Spearman or Pearson correlations, depending on data distribution. Values represent correlation coefficients, with p-values in parentheses. Abbreviations: BMI, body mass index; BRS, baroreflex sensitivity; DBP, diastolic blood pressure; HF, high frequency; HRV, heart rate variability; LF, low frequency; MAP, mean arterial blood pressure; SBP, systolic blood pressure.

|  | **Early (N=16)** | **Late (N=13)** | **p-value** |
| --- | --- | --- | --- |
| **HRV-LF [%]** | 36 ± 15 | 41 ± 22 | 0.549 |
| **HRV-HF [%]** | 14 (11-36) | 18 (9-29) | 0.589 |
| **Sequence-BRS [ms/mmHg]** | 8 ± 4 | 7 ± 3 | 0.627 |
| **Spectral-BRS [ms/mmHg]** | 7 (7-9) | 8 (6-11) | 0.779 |

**Table S2** **–** **Potential confounding effects of testing time on autonomic tone.** Values are presented as mean and standard deviation or median and interquartile range, depending on data distribution. The early group included sessions conducted between 07:00 and 11:00h, whereas the late group comprised sessions performed between 13:00 and 18:30h. Cardiovascular recordings could not be obtained for one participant because anatomical features of the finger prevented adequate placement of the finger cuff. Abbreviations: BRS, baroreflex sensitivity; HF, high frequency; HRV, heart rate variability; LF, low frequency.

| **Pain inhibitory capacity [%]** | **PCS** | **HADS-A** | **HADS-D** |
| --- | --- | --- | --- |
| Conditioned pain modulation (parallel) | -0.18 (0.35) | -0.24 (0.20) | -0.04 (0.83) |
| Conditioned pain modulation (sequential) | 0.08 (0.68) | -0.25 (0.19) | -0.04 (0.84) |
| Offset analgesia | 0.11 (0.56) | 0.07 (0.70) | 0.25 (0.19) |

**Table S3 – Bivariate correlation matrix between pain inhibitory capacity and psychological characteristics.** Values represent Spearman's correlation coefficients (rho) with p-values in parentheses. Abbreviations: HADS-A, hospital anxiety and depression subscale for anxiety; HADS-D, hospital anxiety and depression subscale for depression; PCS, pain catastrophizing score.

|  | **Parallel CPM [%]** | **OA [%]** | **Sequential CPM [%]** |
| --- | --- | --- | --- |
| **HRV-LF [%]** |  |  |  |
| uncorrected p | 0.081 | **0.007** | 0.404 |
| FDR-corrected p | 0.102 | **0.030** | 0.935 |
| **HRV-HF [%]** |  |  |  |
| uncorrected p | 0.988 | 0.418 | 0.601 |
| FDR-corrected p | 0.988 | 0.697 | 0.935 |
| **Sequence-BRS [ms/mmHg]** |  |  |  |
| uncorrected p | **0.048** | 0.996 | 0.790 |
| FDR-corrected p | 0.080 | 0.996 | 0.935 |
| **Spectral-BRS [ms/mmHg]** |  |  |  |
| uncorrected p | **0.006** | **0.012** | 0.935 |
| FDR-corrected p | **0.021** | **0.030** | 0.935 |
| **SBP reactivity [%]** |  |  |  |
| uncorrected p | **0.008** | 0.930 | **0.007** |
| FDR-corrected p | **0.021** | 0.996 | **0.033** |
| **HRV-RMSSD [ms]** |  |  |  |
| uncorrected p | **0.038** | 0.722 |  |
| FDR-corrected p | **0.038** | 0.722 |  |
| **HRV-SDRR [ms]** |  |  |  |
| uncorrected p | **0.005** | 0.277 |  |
| FDR-corrected p | **0.011** | 0.553 |  |

**Table S4 –** **Uncorrected and corrected p-values of all correlations.** This table presents both uncorrected and multiple-comparison-corrected p-values for all correlation analyses. All reported correlations are already adjusted for previously identified confounding factors. Significant results are highlighted in bold. Abbreviations: BRS, baroreflex sensitivity; CPM, conditioned pain modulation; HF, high frequency; HRV, heart rate variability; LF, low frequency; OA, offset analgesia; RMSSD, root mean square of successive R-R interval differences; SBP, systolic blood pressure; SDRR, standard deviation of successive R-R interval.

Figure S1 – Partial correlation analyses between sequential CPM capacity and heart rate variability, baroreflex sensitivity and blood pressure reactivity. Negative values for CPM capacity depict pain inhibition, while positive values show pain facilitation. Significant correlations are illustrated by a green regression line and non-significant correlations by a black regression line. Partial correlations between CPM capacity and (a) HRV-LF power adjusted for sex, (b) HRV-HF power adjusted for sex, (c) sequence-BRS adjusted for baseline SBP, (d) spectral-BRS adjusted for baseline SBP and sex, and (e) SBP reactivity. P-values were corrected using a Benjamini-Hochberg correction for a family of five tests. Abbreviations: BRS, baroreflex sensitivity; CPM, conditioned pain modulation; HF, high frequency; HRV, heart rate variability; LF, low frequency; SBP, systolic blood pressure.

Figure S2 – Partial correlation analyses between endogenous pain modulation and time-domain HRV parameters. Negative values for endogenous pain modulation depict pain inhibition, while positive values show pain facilitation. Significant correlations are illustrated by a green regression line and non-significant correlations by a black regression line. Partial correlations between parallel CPM capacity and (a) RMSSD adjusted for sex and BMI, (b) SDRR adjusted for sex, age and BMI; and between OA capacity and (c) RMSSD adjusted for sex and BMI, and (d) SDRR adjusted for sex, age and BMI. P-values were corrected using a Benjamini-Hochberg correction for a family of two tests for each paradigm (i.e., CPM and OA). Abbreviations: BMI, body mass index; CPM, conditioned pain modulation; OA, offset analgesia; RMSSD, root mean square of successive R-R interval differences; SDRR, standard deviation of successive R-R interval.
